# Congenital combined venolymphatic malformation (ISSVA classification VM-LM): a rare clinical image

**DOI:** 10.11604/pamj.2024.47.155.43020

**Published:** 2024-04-02

**Authors:** Shraddha Patil, Sonali Kolhekar

**Affiliations:** 1 Department of Child Health Nursing, Smt. Radhikabai Meghe Memorial College of Nursing, Datta Meghe Institute of Higher Education and Research (Deemed University) Sawangi (Meghe) Wardha, Maharashtra, India

**Keywords:** Vascular anomalies, lymphatic malformation, venous malformation, sclerotherapy

## Image in medicine

Venolymphatic malformations (VLMs) are uncommon congenital defects characterized by abnormal venous and lymphatic channel development. Venolymphatic malformation is a congenital defect that appears on the flexor aspect of the elbow at birth. A 7-year-old male child came to a rural hospital with care of swelling over the right elbow. As narrated by the father, the child has had swelling over the forearm since birth. The swelling was initially small in size, but gradually progressed to the current size (A). The child was taken to a local hospital where symptomatic treatment was given. The child was advised for Fine Needle Aspiration Cytology (FNAC) of which finding shows single scaper spindle-shaped cells along with cyst macrophages against the hemorrhagic background. On X-ray, the upper forearm shows soft tissue swelling with ill-defined radio densities at the soft tissue plane (B). Magnetic Resonance Imaging (MRI) examinations reveal flow vascular malformations such as venous malformation/ hemangioma in the forearm and arm (C). An MRI of the brain was performed, and the results were normal. On the ultrasound examination, a significant well-defined, solid, hypoechoic lesion is shown in the intramuscular plane of the right arm's biceps muscle (2.7 x 2.3 x 2.3 cm) (D). A few small cystic areas were noted within the lesion. Minimal vascularity was seen in the lesion with significant peripheral vascularity. There are multiple duct-like structures seen in the right forearm swelling below the posterior aspect elbow. The child was brought for re-embolisation sclerotherapy done at the level of the elbow with sethol and bleomycin and contrast +foam +air had been instilled. The second pocket has been closed and the procedure was uneventful. Patients with venolymphatic malformations are managed with observation, surgical excision, and sclerotherapy. Long-term follow-up is essential for individuals with congenital combined vascular venolymphatic malformation to detect any changes in symptoms or consequences. This may involve regular clinical evaluations, imaging studies, and adjustments to treatment as needed. Additionally, patient education and support are essential to help individuals manage their condition and optimize their quality of life.

**Figure 1:**
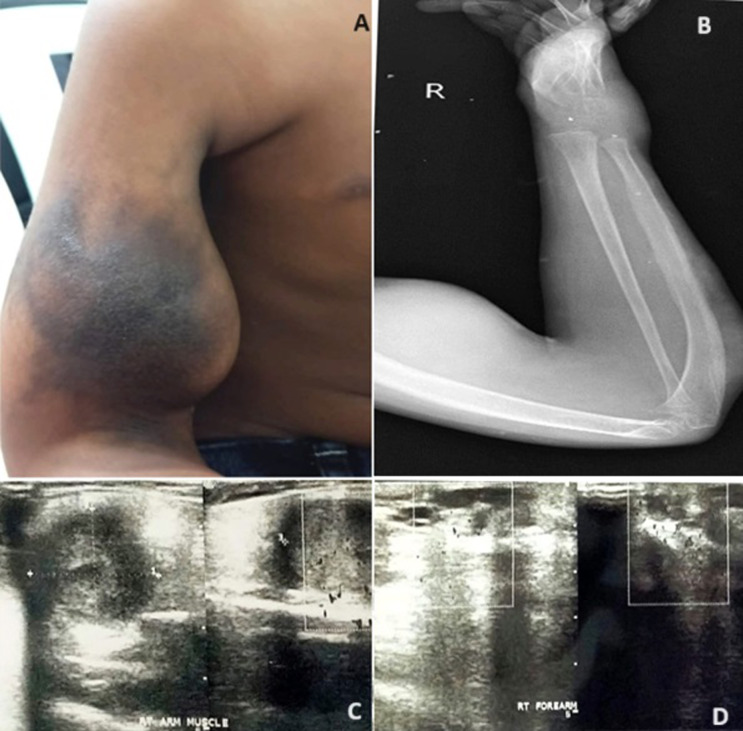
A) vascular malformation on right elbow blackish patch; B) X-ray of the upper forearm shows soft tissue swelling with ill-defined radio densities at the soft tissue plane; C) multiple duct-like structure seen in the right forearm swelling below the posterior aspect elbow; D) large well defined, solid, hypoechoic lesion seen in the intramuscular plane of biceps muscle of right arm (size: 2.7 X 2.3 X 2.3 cm)

